# Probing microstructural changes in muscles of leptin-deficient zebrafish by non-invasive *ex-vivo* magnetic resonance microimaging

**DOI:** 10.1371/journal.pone.0284215

**Published:** 2023-04-14

**Authors:** Muhamed N. Hashem Eeza, Rico Singer, Yi Ding, Junling He, Zain Zuberi, Hans J. Baelde, Huub J. M. de Groot, Jörg Matysik, Herman P. Spaink, A. Alia

**Affiliations:** 1 Institute of Medical Physics and Biophysics, Leipzig University, Leipzig, Germany; 2 Institut für Analytische Chemie, Leipzig University, Leipzig, Germany; 3 Leiden Institute of Chemistry, Leiden University, Leiden, The Netherlands; 4 Institute of Biology (IBL), Leiden University, Leiden, The Netherlands; 5 Department of Pathology, Leiden University Medical Center, Leiden, The Netherlands; University of Rochester, UNITED STATES

## Abstract

Leptin is a hormone that plays a key role in controlling food intake and energy homeostasis. Skeletal muscle is an important target for leptin and recent studies have shown that leptin deficiency may lead to muscular atrophy. However, leptin deficiency-induced structural changes in muscles are poorly understood. The zebrafish has emerged as an excellent model organism for studies of vertebrate diseases and hormone response mechanisms. In this study, we explored *ex-vivo* magnetic resonance microimaging (μMRI) methods to non-invasively assess muscle wasting in leptin-deficient (*lepb*^*-/-*^) zebrafish model. The fat mapping performed by using chemical shift selective imaging shows significant fat infiltration in muscles of *lepb*^*-/-*^ zebrafish compared to control zebrafish. *T*_*2*_ relaxation measurements show considerably longer *T*_*2*_ values in the muscle of *lepb*^*-/-*^ zebrafish. Multiexponential *T*_*2*_ analysis detected a significantly higher value and magnitude of long *T*_*2*_ component in the muscles of *lepb*^*-/-*^ as compared to control zebrafish. For further zooming into the microstructural changes, we applied diffusion-weighted MRI. The results show a significant decrease in the apparent diffusion coefficient indicating increased constraints of molecular movements within the muscle regions of *lepb*^*-/-*^ zebrafish. The use of the phasor transformation for the separation of diffusion-weighted decay signals showed a bi-component diffusion system which allows us to estimate each fraction on a voxel-wise basis. A substantial difference was found between the ratio of two components in *lepb*^*-/-*^ and control zebrafish muscles, indicating alterations in diffusion behavior associated with the tissue microstructural changes in muscles of *lepb*^*-/-*^ zebrafish as compared to control zebrafish. Taken together, our results demonstrate that the muscles of *lepb*^*-/-*^ zebrafish undergo significant fat infiltration and microstructural changes leading to muscle wasting. This study also demonstrates that μMRI provides excellent means to non-invasively study the microstructural changes in the muscles of the zebrafish model.

## Introduction

Leptin is an adipocytokine composed of 167 amino acids [1] produced mainly by mature adipocytes in white adipose tissue and encoded by the Ob gene [2, 3]. Leptin is considered pleiotropic in mammals, as it plays a key role in food intake, energy homeostasis, and lipid utilization by regulating behavioral, autonomic, and endocrine circuits in the central nervous system (CNS) to control energy intake and expenditure and maintaining the long-term fat stores [4, 5]. Insufficient leptin signaling in mammals leads to obesity, diabetes, and hyperphagia [6]. Skeletal muscle is considered to be directly affected by leptin in terms of its function in regulating glucose metabolism [1]. Leptin receptors are abundant in skeletal muscle and leptin has a role of boosting glucose utilization and fatty acid metabolism in skeletal muscles in both animal models and humans [5, 7, 8]. Studies in mice and rats have shown that leptin promotes glucose uptake in skeletal muscle via the hypothalamic–sympathetic nervous system axis, and it stimulates the oxidation of fatty acid in muscle via AMP-activated protein kinase (AMPK) [8]. Thus, Leptin has an important role in promoting muscle growth and decreasing muscle atrophy [9, 10].

In humans, during aging, a reduction in the circulating levels of leptin has been shown to be associated with muscle atrophy [11, 12]. In leptin-deficient ob/ob mice, lack of leptin has been found to be associated with muscular atrophy, and reduced muscle growth and muscle mass [7, 9]. Leptin treatment has been shown to increase muscle mass and reduce muscle wasting in ob/ob mice [9, 12]. Leptin was recently approved by the Food and Drug Administration (FDA) for the treatment of lipodystrophy [13], considering that leptin treatment may enhance positive regulators of muscle cell proliferation [9, 12, 14] along with correcting infertility, diabetes, and immune abnormalities [13]. Since leptin promotes energy dissipation and prevents fatty acid accumulation the deficiency of leptin may result in intramuscular accumulation of lipids in muscle. However, studies on the impact of leptin deficiency on muscle adiposity are lacking. Moreover, probing microstructural changes in muscle would be necessary to get a better understanding of muscle wasting during leptin deficiency.

In recent years, zebrafish has been firmly established as an ideal model organism for studies of vertebrate diseases, biological pathways, and hormone response mechanisms. Several studies suggest that zebrafish have similar muscle structure and development to that of mammals, and that the majority of muscle-related genes tested are present in zebrafish [15–18]. With regard to leptin, zebrafish have two major leptin genes: *lepa* and *lepb*, and one leptin receptor gene *lepr* [19, 20]. Previously, the obesity phenotype has been observed in a *lepa*-deficient adult zebrafish [21], however in another study, the obesity phenotype was not consistently observed in *lepa or lepr* deficient zebrafish [22]. Recently, Kamstra *et al*. have shown that overfeeding elicits elevated body weight in both *lepr and lepa* deficient zebrafish as compared to control [23]. They have further provide evidence that leptin regulates glucose homeostasis via the wingless/integration1 (Wnt) pathway [23].

We have recently generated *lepb*-deficient (*lepb*^*-/-*^) adult zebrafish via a CRISPR-CAS9 gene editing approach [24]. The deletion of *lepb* gene resulted in the development of type 2 diabetes mellitus (T2DM) and diabetic complications. Furthermore, we observed that *lepb*^*-/-*^ zebrafish had an increase in body weight and visceral fat accumulation [24]. Our results demonstrated that *lepb* regulates glucose homeostasis and adiposity in zebrafish. Using this interesting model, in this work, we want to address how leptin deficiency contributes to atrophy and adiposity more specifically in muscles. We used state-of-the-art magnetic resonance imaging methods to probe microstructural changes in the muscles of *lepb*^*-/-*^ zebrafish *ex-vivo*, to address this question non-invasively. Chemical shift selective imaging methods were used to selectively follow fat infiltration in the muscles of *lepb*^*-/-*^ zebrafish. *T*_*2*_ relaxation measurements of zebrafish muscles as well as *T*_*2*_ multicomponent analysis were used to obtain microstructural changes and quantitative information about water compartments in the muscle of *lepb*^*-/-*^ zebrafish. In addition, we utilized diffusion MRI along with the phasor transformation approach of diffusion data to follow alterations in diffusion behaviour associated with the tissue microstructural changes in muscles of *lepb*^*-/-*^ zebrafish.

## Materials & methods

### Maintenance of zebrafish

In this study, we utilized wild-type (wt) AB/TL strain (control) and homozygous leptin receptor b knockdown mutant (*lepb*^*-/-*^). Homozygous F1 carriers were out-crossed once against wt, and were subsequently in-crossed, resulting in *lepb*^*-/-*^ and *lep*^*+/+*^ siblings. The *lepb*^*-/-*^siblings were used for experiments described in this work. For genotyping and selecting *lepb*^*-/-*^ sibling, genomic DNA was amplified using forward primer 5′-GAGACTCTCCTGAGGACACTGG-3′ and reverse primer 5′-GCATGGCTTACACATTTCAGAG-3′, amplifying a 201 base pair (bp) product containing the mutation, which can be detected using 2% agarose gel. Both control and leptin mutant were kept in recirculating aquaria of size (L 80 cm; H 50 cm; W 46 cm) holding no more than 60 in number. The flow rate of water was 150 L/min and the temperature was maintained at 28±0.5°C. To mimic day and night cycles, the light was exposed for 14 h and was kept in dark for 10 h. Spirulina brand flake food (O.S.L. Marine Lab., Inc., Burlingame, USA) was fed twice daily and twice a week with frozen food (Dutch Select Food, Aquadistri BV, the Netherlands). Zebrafish strains were handled in compliance with the local animal welfare regulations and maintained according to standard protocols (zfin.org). The use of adult zebrafish was approved by the local animal welfare committee (DEC) of the University of Leiden (license number: AVD1060020171767) and adhered to the international guidelines specified by the EU Animal Protection Directive 2010/63/EU. For imaging, adult zebrafish (aged between 4 and 6 months) were euthanized through immobilization by submersion in ice water (0-4°C) for at least 10 minutes following cessation of opercular movement and then fixed in 4% buffered paraformaldehyde (Zinc Formal-Fixx, ThermoShandon, UK) for 4 days and subsequently embedded in Fomblin (perfluoropolyether) for MRI measurements. Control (N = 12) and leptin-deficient (N = 12) zebrafish of both sex were used in this study.

### MRI experiments

MR imaging scans were performed using 300 MHz (7T) or 750 MHz (17.6T) Bruker vertical bore system (Bruker Biospin GmbH, Germany). A birdcage transmit/receive radiofrequency (RF) coil with an inner diameter of 10 mm was utilized at both magnets. The workstation was interfaced with a Linux operating system running ParaVision 5.1 (at 7T) or ParaVision 360 v3.3 (at 17.6T) imaging software (Bruker Biospin GmbH, Germany). Prior to each measurement, the magnetic field homogeneity was optimized by shimming the magnet, followed by the frequency calibration, transmitter gain, and receiver gain adjustments. Each session of measurements started with a multislice orthogonal gradient-echo sequence to determine the position and select the desired region for subsequent experiments.

For anatomical imaging of zebrafish, a rapid acquisition with relaxation enhancement (RARE) sequences was used [25]. RARE is a fast imaging sequence that employs an RF excitation pulse followed by a train of refocusing pulses to produce multiple RF spin echoes that speed up image acquisition by acquiring more than one *k*-space line per repetition [26]. Basic measurement parameters used for the RARE sequence were: Echo time TE = 15 ms with an effective echo time of 33.6 ms; Repetition time (TR) = 2000 ms; Number of scans (ns) = 8; Total scan time = 17 min. RARE factor = 4; The field of view (FOV) was 1.5 cm with a matrix size (MS) of 256 x 256; The slice thickness was 0.5 mm.

Chemical shift selective (CHESS) imaging scheme, which utilizes this principle of chemical shift, was used to acquire selective fat images [27]. The CHESS sequence is composed of a single frequency-selective excitation pulse with a flip angle of π/2 followed by a dephasing gradient (homogeneity spoiling gradient). The procedure leaves the spin system in a state where no net magnetization of the unwanted component is retained while the wanted component remains entirely unaffected in the form of z-magnetization [27]. On-resonance frequency-selective excitation was performed using a 90° Gaussian pulse with a narrow bandwidth. Measurement parameters of CHESS were: TE = 6 ms with an effective echo time of 13.3 ms; TR = 800 ms; Number of scans (ns) = 12; Total scan time = 28 min. The field of view (FOV) was 1.5 cm with a matrix size (MS) of 256 x 256; The slice thickness was 0.5 mm and the interslice distance was also 0.5 mm.

Chemical Shift Imaging (CSI) was applied in spin echo slab selective mode to obtain multivoxel spectroscopic information. Instead of a conventional readout gradient used in imaging, CSI employs two orthogonal phase encoding steps with pulsed gradients to record a pure spectroscopic echo during acquisition. Simultaneous spatial and spectral resolution enables multi-voxel spectra and the outcome is a form of maps representing the spatial distribution of individual metabolites [28, 29]. For an improved Spatial Response Function (SRF), a Hanning function weighted *k*-space acquisition scheme was utilized, as implemented by the Bruker *‘weighted’* measuring method. Basic measurement parameters were as follows: TE = 15 ms; TR = 3500 ms; Matrix = 32x32; FOV = 20x20 mm^2^; slice thickness = 2 mm; resolution = 1.25x1.25x2 mm^3^; number of scans = 256. For display purpose, the data was reconstructed into a 64x64 matrix with linear smoothing. Sinc3 pulses with a bandwidth of 8000 Hz were used for excitation and refocusing. The echoes were captured into 2048 points over 204.80 ms, resulting in a spectral resolution of 2.4 Hz per point; and a spectral width of 10 kHz (13.3 ppm). Optimization of the magnetic field homogeneity in the selected volume was achieved by shimming the water resonance. For efficient water signal saturation, a VAPOR suppression scheme with a duration of 625 ms was applied. Seven Hermite-shaped CSI modules were used, with interpulse radiofrequency delays of 150, 80, 160, 80, 100, 37.2 and 15 ms. The RF bandwidth was 900 Hz and the excitation offset was set at -75 Hz (-0.1 ppm).

The *T*_*2*_ relaxation times were determined with a standard multi-slice multi-echo (MSME) sequence that employs the Carr-Purcell Meiboom-Gill (CPMG) pulse sequence, where 90° excitation pulse is refocused by a series of 180° pulses generating a series of echos [30, 31]. A minimum echo time of 5.24 msec was used to allow for more echo images to be acquired within one cycle to achieve robust *T*_*2*_ fitting [32]. The following imaging parameters were used: TR = 2500 msec, nominal flip angles = 90° and 180°, and a train of 30 echoes with TEs ranging from 5.24 msec to 157.20 msec with 5.24 msec echo-spacing, the number of scans was 4 (total measurement time 42.6 min). The field of view was equal to 19.0 × 36.0 mm^2^, with an in-plane resolution of 74 μm^2^/pixel^2^. Four slices of 0.2 mm thickness with a 0.2 mm interslice distance were acquired. The *T*_*2*_ measurement using a multi-echo spin sequence can be influenced by the magnetization transfer in a multislice acquisition [33]. To rule out the biased signal due to magnetization transfer and corresponding influence on *T*_*2*_ measurement, we performed multi-echo spin acquisition on a control fish using a single slice (0.2 mm slice thickness) vs multislice (4 slices; 0.2 mm thickness and with a 0.2 mm interslice distance). No significant difference was seen in *T*_*2*_ quantification using single vs four slice acquisition (S1 Table in S1 File).

Diffusion measurements were performed at 17.6 T using a spin-echo pulse sequence with a pair of mono-polar diffusion-sensitizing gradients [34]. Gradient orientations were evenly distributed in six isotropic directions. Effective B-values range: 221, 267, 399, 614, 914, 1299, 1792, 2385, 3070, 3846, 4714 s/mm^2^. A diffusion gradient duration (δ) of 4 ms was utilized in conjunction with a 50 ms diffusion gradient separation (Δ), resulting in a total TR and TE of 1000 and 60.2 ms, respectively. In order to achieve sufficient SNR, two averages were acquired, resulting in an overall acquisition time of 8.5 hours. The FOV was 20x20 mm^2^, matrix size 256x256 and the slice thickness was set to 1 mm, resulting in a spatial resolution of 78x78x1000 μm^3^.

### Data processing

#### Quantitative analysis of fat in CHESS images

For quantitative analysis of fat in CHESS images, the image slices were exported and analyzed in ImageJ software. By using a plugin, the area of the fish was defined, and a certain threshold was adjusted to eliminate any contribution of noise. Subsequently, the hyperintense signal of fat was calculated. The data were exported to Origin Pro v. 8 software for further analysis.

#### Estimation of T_2_

For the calculation of *T*_*2*_ relaxation time, regions of interest (ROIs) were drawn at various locations within the muscle region using an image sequence analysis (ISA) tool package (Paravision 5, Bruker). A monoexponential fitting approach was employed to calculate *T*_*2*_ values, using a monoexponential fit function

y=A+C*exp(–t/T2) (Eq 1)

where A = Absolute bias, C = signal intensity, *T*_*2*_ = transverse relaxation time. Means and standard deviation for *T*_*2*_ relaxation times for each ROI were calculated.

#### T_2_ Multicomponent analysis

Tools were developed using MATLAB (The Mathworks Inc, Natick, MA) to perform multicomponent *T*_*2*_ analysis, using the curve fitting (CF) tool as described earlier [35]. The *T*_*2*_ decay curve was fitted to a multi-component *T*_*2*_ model, as shown in the equation below. To decompose the decay curves into multiple exponential components, the nonnegative least-squares (NNLS) algorithm was utilized [35, 36]. The NNLS algorithm was executed using MATLAB codes and the “Isqnonlin” function was employed to analyze the optimal solution of the data from imaging experiments. The signal intensities, in MRI methods, as a function of the echo time can be written as:

$$y(t_{i})=\sum_{i=1}^{M}S_{i}e^{-t_{i}/T_{2i}}+C,i=1,2,\ldots ,N$$ (Eq 2)

Here, N represents the number of echoes, *t*_*i*_ is the *ith* echo time, *y*(*t*_*i*_) is the signal intensity of the *ith* echo, *M* denotes the number of *T*_*2*_ components, *S*_*i*_ represents the intensity of the *ith T*_*2*_ component, and C is a constant that account for any signal offset. To generate both the simulation and experimental data, a train of 30 echoes (M = 30) was employed. An extra *T*_*2*_ value, which was set to infinity, was included to simulate the constant item in equation (Eq 1). To produce a *T*_*2*_ distribution, a “least-square based constraint” rule was applied. During the calculation, the regularization value was selected in a manner that ensured the estimated *T*_*2*_ distribution was consistent with the known *T*_*2*_ distribution for experimentally calculated data. From the NNLS calculation, any *T*_*2*_ component with an intensity below a specific threshold (peak area less than 0.1% of the total area) or with the *T*_*2*_ value at the peak position lower than 0.5ms or higher than 300 ms threshold was ignored from each *T*_*2*_ spectrum. The NNLS procedure used in this work implies that the results were acquired without making any prior assumptions regarding the number of *T*_*2*_ components or an initial estimate of the solution.

#### Processing of diffusion data

Diffusion data is analysed by the phasor approach [37] using MATLAB 2021b (Mathworks, Natick, MA, USA). Raw data is imported via a modified version of read_2dseq and transformed to phasor plot coordinates by data selection, filtering by an Arithmetic Mean Filter, normalization, and applying Fast-Fourier Transformation (MATLAB’s fft function), harmonic selection, and plotting real versus imaginary part. Finally, the phasor plot semi-circle is added for apparent diffusion coefficient (ADC) values between 0 and 0.1 mm^2^/sec. Mono-component analysis of diffusion data is performed by projecting the phasor plot coordinate of selected voxels onto the semi-circle by MATLABs dsearchn() function. ADC components for bi-component analysis are determined by fitting the line created between two ADC values on the semi-circle through the phasor plot coordinates. As a control, the fit (R^2^) of the found ADCbi values is determined by the curve fitting tool of MATLAB using the description for diffusion;

s(b)=a.exp(−b.ADC1)+c.exp(−b.ADC2)+d (Eq 3)

where b refers to b-values, a and c to the relative abundance of each ADC component, and d to a constant. For every voxel, the contribution ratio of ADCbi 1 / ADCbi 2 is calculated by projecting phasor plot coordinates onto the fictional line created by the ADC components. Statistical analysis to compare *lepb*^*-/-*^ zebrafish to controls was performed by two-sample t-test (MATLABs ttest2() function) and p-values above the 95% threshold (p < 0.05) were considered significant.

#### Histology

The adult zebrafish were fixed for one week in 4% PFA at 4°C. Subsequently, they were washed twice with PBS and then were transferred to EDTA (100mM, pH = 8) for decalcification at room temperature for another 1 week, followed by embedding in paraffin. The slices through the muscle areas were cut (4-μm thickness) using a Leica microtome (Wetzlar) and then slices were stained with hematoxylin and eosin (HE) using standard protocols.

#### Statistical analyses

All statistical analysis of the data was performed using OriginPro v. 8 (Northampton, USA). One-way analysis of variance (ANOVA) with the Bonferroni test for comparison of mean between two groups was performed. A p-value of <0.05 was considered significant.

## Results & discussion

**Fig 1:**
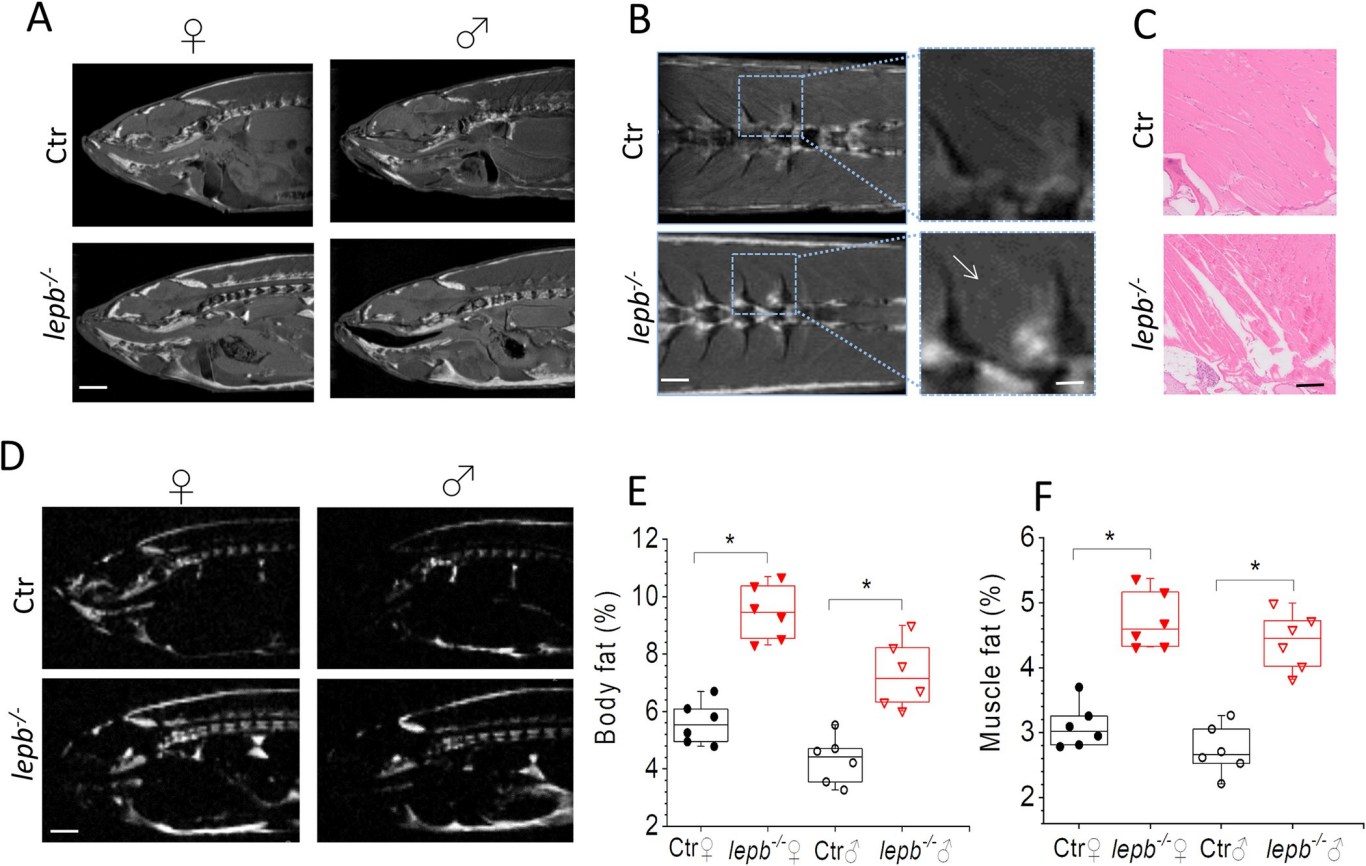
Magnetic resonance anatomical imaging and selective fat imaging in control (Ctr) and *lepb*-deficient (*lepb*^*-/-*^) adult zebrafish. (A) Representative sagittal MR images of female (♀) and male (♂) Ctr and *lepb*^*-/-*^ zebrafish measured by RARE pulse sequence (TE = 15 ms with effective TE of 33.6 ms; TR = 2000 ms; ns = 8; Scan time 17 min). Scale bar: 1 mm. (B) Representative coronal MR images showing muscle area of female Ctr and *lepb*^*-/-*^ zebrafish measured by RARE pulse sequence. Scale bar: 500 μm. A zoomed view (right column) showing fat infiltration in muscles of *lepb*^*-/-*^ zebrafish. Scale bar: 250 μm; (C) Histological sections from the same muscle region as in (B) stained with HE staining. Scale bar: 250 μm. (D) Images of fat distribution in the sagittal plane, acquired with Chemical Shift Selective (CHESS) pulse sequence in female Ctr and *lepb*^*-/-*^ zebrafish. Scale bar: 1 mm; (E) Quantification of body fat in control and *lepb*^-/-^ female and male adult zebrafish measured from CHESS MR images (n = 6 per group) (***p < 0.001); (F) Quantification of fat in muscle region from control and *lepb*^-/-^ female and male adult zebrafish measured from CHESS MR images (n = 6 per group) (***p < 0.001).

In this study, we probed the effect of leptin deficiency on the microstructural changes in the muscle of *lepb*^*-/-*^ zebrafish model using state-of-the-art MR imaging, which enabled us to non-invasively analyze the muscle wasting and fat infiltration caused by the deficiency of leptin. High-resolution anatomical images of control (Ctr) and leptin-deficient (*lepb*^*-/-*^) male and female zebrafish are displayed in Fig 1A. An increased fat content can be seen in the body of *lepb*^*-/-*^ zebrafish of both sex, including noticeable visceral fat accumulation and, most importantly, fat infiltration in and around the skeletal muscles. This can be clearly seen in Fig 1B, which represents a zoomed-in image of the muscle fiber from coronal slices, where the distribution of fat inside the muscle tissue of *lepb*^*-/-*^ is more clearly noticed in comparison to control fish. Histological examination of slices from the same region, stained with hematoxylin and eosin (HE), show a normal architecture of muscle tissues in the control fish and substantial wasting of the skeletal muscles fibers of *lepb*^*-/-*^ fish which can be described as atrophic myocytes and/or myocyte losses (Fig 1C). Previous studies in human have shown that low circulating levels of leptin are associated with muscle atrophy [11, 12]. Similarly, in leptin-deficient ob/ob mice, muscular atrophy, reduced muscle growth, and muscle mass was observed [9]. Our results show that leptin deficiency in zebrafish has a similar effect on muscle atrophy, and further muscle adiposity which may have been associated with an imbalance of glucose utilization and fatty acid metabolism in skeletal muscles.

**Fig 2:**
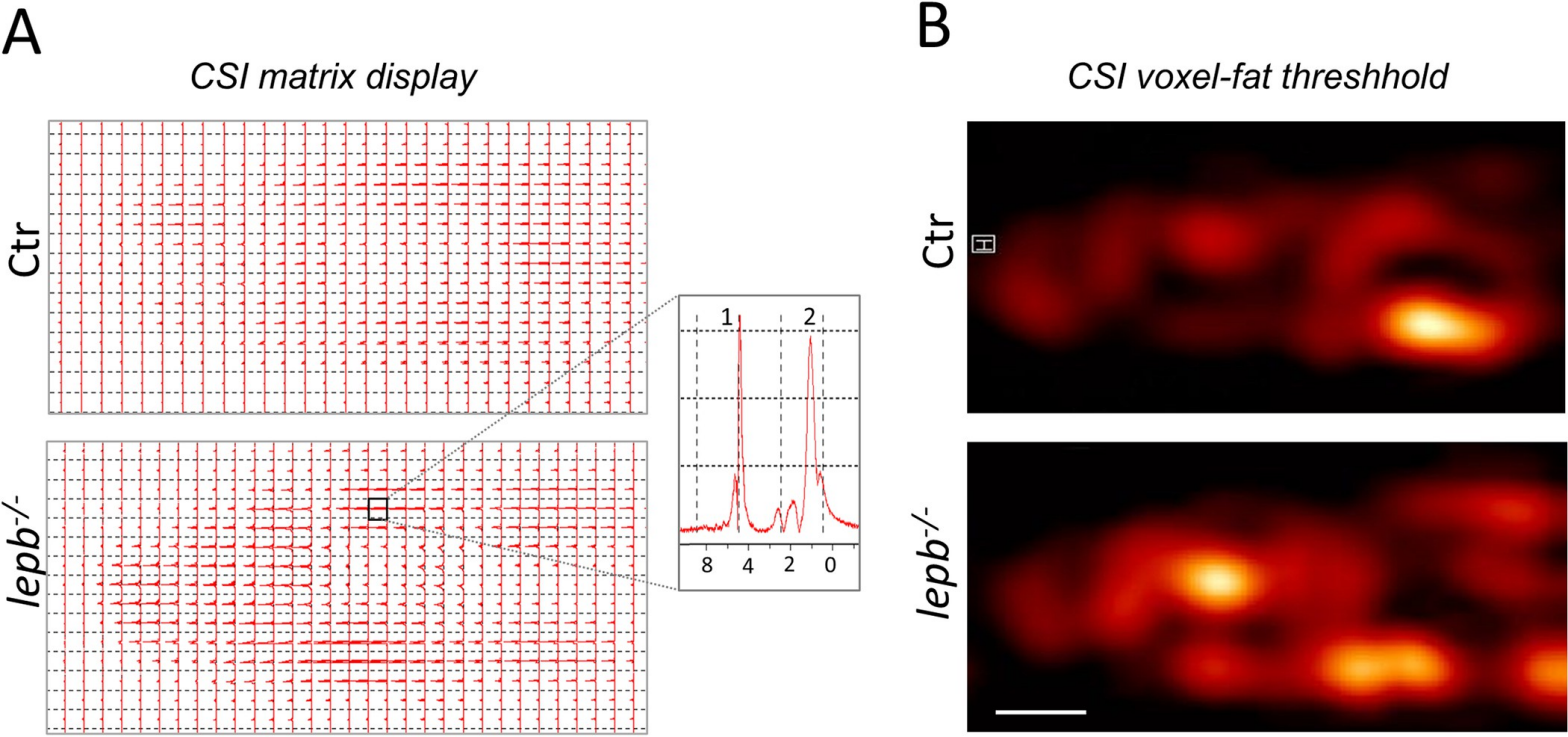
Chemical shift imaging. (A) Matrix display of chemical shift imaging spectra. CSI data was recorded with a TR = 3500 ms; TE = 15 ms and slice thickness was 2 mm. Total averages were 256. The resolution obtained was 1.25×1.25×2 mm^3^. The spectral width used was 10 kHz (13.33 ppm) and 32x32 matrix was reconstructed into 64×64 voxels. Inset: Representative spectra of a single voxel showing residual water (1) and fat resonances (2). The main–(CH_2_)_n_−signal in muscles is centered around 1.3 ppm, with side lobes from–(CH_2_)_n_–CH_3_ up-field and -CH_2_-CH = CH-,–CH_2_–CH_2_–COOR extending downfield. (B) CSI voxel intensity thresholding. Signals between 0.80 to 1.25 ppm corresponding to fat were chosen to reconstruct CSI images and they were overlaid with corresponding *T*_*2*_-weighted RARE images using the Bruker CSI Visualisation Tool. Scale bar: 1 mm.

In order to exclusively image fat distribution, we have utilized the chemical shift selective (CHESS) pulse sequence to resolve the spatial distribution of fat in and around the skeletal muscles. As shown from CHESS images in Fig 1D, where an increased amount of fat infiltrated in the muscles can be seen of *lepb*^*-/-*^ in comparison to those of control fish. To improve image quality and to further confirm fat infiltration, we also imaged the *lepb*^*-/-*^ and control fish at an ultra-high magnetic field (17.6T) with and without fat suppression. The improved image quality in the muscle areas was observed at 17.6T as characterized by a better signal-to-noise (SNR), better image contrast, and higher resolution (S1A Fig in S1 File). As clear from this figure, significant fat infiltration was observed in various regions including the muscle areas of *lepb*^*-/-*^ zebrafish, which was not seen in images obtained through fat suppressed RARE sequence (S1B Fig in S1 File). Fat signal which was selectively obtained by a 3D chemical shift selected imaging sequence (red color) is overlayed on 3D RARE (grayscale) image, which is shown in S1C Fig in S1 File. A quantitative analysis of body fat and muscles fat obtained from CHESS images is shown in Fig 1E, 1F. It can be clearly observed that the body fat, as well as muscle fat percentage, is significantly higher in *lepb*^*-/-*^. Non-invasive chemical shift imaging (CSI) was utilized to further analyse the content and distribution of fat spectroscopically. CSI allows mapping the spatial distribution of hydrogen nuclei associated with water or with lipid molecules. Fig 2A displays CSI data in the form of a matrix array of spectra. A representative spectrum, taken from the highlighted region of Fig 2A, illustrates two prominent peaks attributed to water (1) and fat (2) within the muscle (inset Fig 2A). The primary signal of fat in muscles, which corresponds to the–(CH_2_)_n_−group is centred at approximately 1.3 ppm. A color map of fat is displayed and overlaid on an MR image of the same slice in Fig 2B. The CSI image of the fat signal revealed that indeed *lepb*^*-/-*^ fish contain a high amount of fat at various locations including muscles as compared to control fish. Thus, the utilization of CSI not only enabled the precise measurement of fat protons but also allowed for the assessment of its distribution and relative intensity in localized domains in *lepb*^*-/-*^ muscles. Taken together, our results show that muscles in *lepb*^*-/-*^ fish contain higher fat concentration as compared to control fish as seen in both CHESS and CSI measurements.

To analyze microstructural changes in muscles non-invasively, we used quantitative transverse relaxation time (*T*_*2*_) measurements. Quantitative *T*_*2*_ measurements have been utilized in previous studies for the assessments of normal and a variety of diseases including characterizing muscle injury and repair [38–42]. *T*_*2*_ relaxation times were measured with the Multi-Slice, Multi-Echo (MSME) sequence. To rule out any influence of magnetic field disturbance on *T*_*2*_ changes measured by MSME sequence, the effect of CPMG refocusing interpulse interval (τ) on *T*_*2*_ for the muscle regions was examined as described earlier [35]. No statistically significant impact of the interpulse interval in the range of interest (6.6 and 12 ms) was observed on the *T*_*2*_ of the muscle areas. This finding indicates that *T*_*2*_ values in muscle regions are primarily influenced by tissue properties rather than magnetic field disturbances.

**Fig 3:**
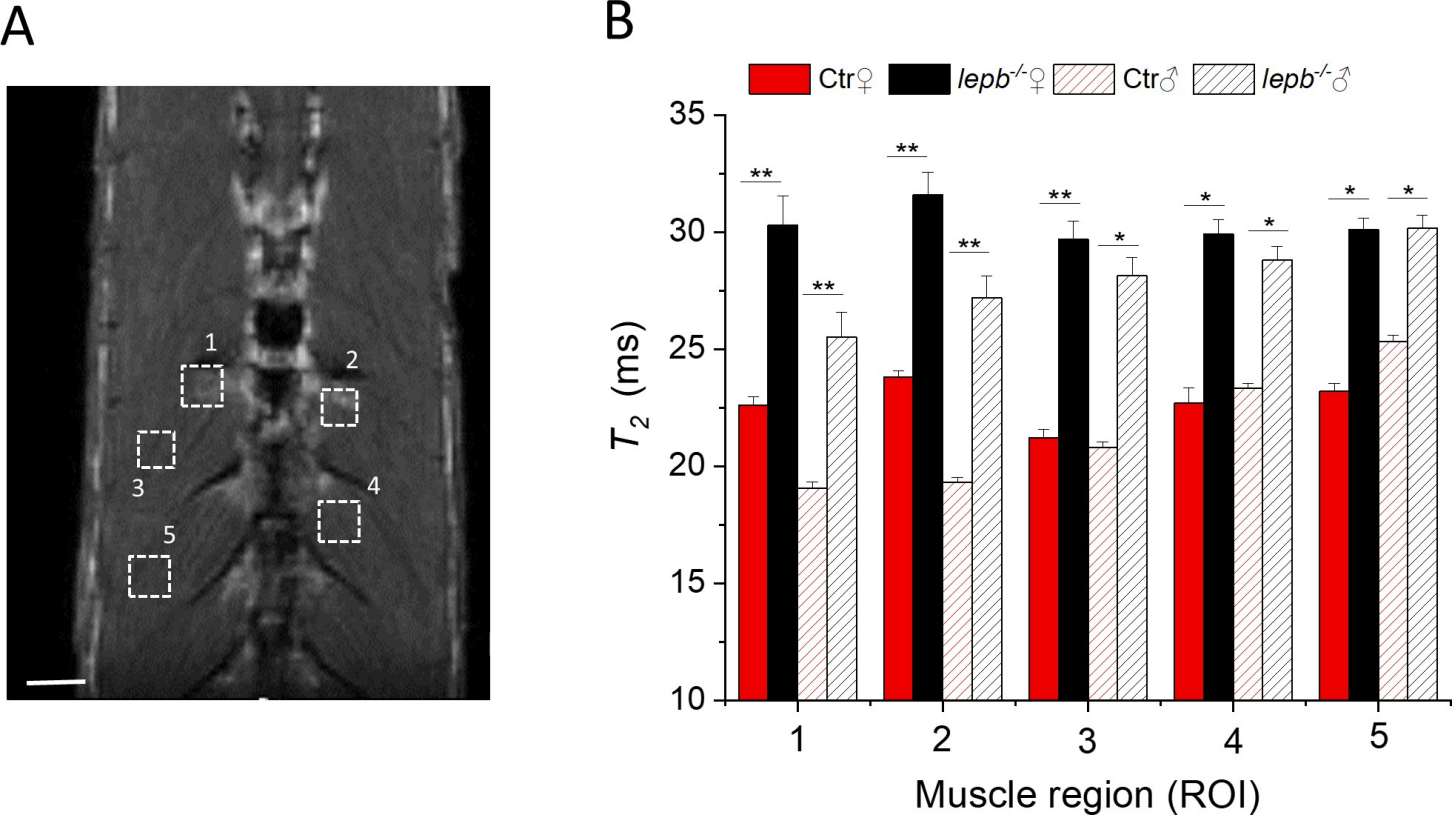
*T*_*2*_ relaxation time measurements in various muscle regions of control (Ctr) and *lepb*-deficient (*lepb*^*-/-*^) adult zebrafish. (A) Anatomical *T*_*2*_ –weighted MR coronal slices of an adult zebrafish, showing various muscle regions for *T*_*2*_ measurements. Scale bar, 500 μm. (B) *T*_*2*_ relaxation time values were measured in five different muscle regions of female (♀) and male (♂) control (Ctr) and leptin-deficient (*lepb*^*-/-*^) zebrafish. The reliability test of *T*_*2*_ measurements was performed by Bonferroni test using a 1-way analysis of variance (ANOVA) to accomplish pairwise comparisons of the data. At the 0.05 level, the *T*_*2*_ population means of *lepb*^*-/-*^ and Ctr are significantly different for both male and female zebrafish. Data represent the mean *T*_*2*_ in ms ± standard deviation (SD) (Error bars) (95% C.I.); n = 6 per group; *p<0.05, **p<0.01.

Fig 3 shows *T*_*2*_ relaxation time changes in five different areas in skeletal muscles (ROI 1–5) of control and *lepb*^*-/-*^ zebrafish. The first two regions of interest (ROI 1–2) were selected in the muscles where significant fat infiltration was visible in both RARE and CHESS images, while the other three regions (ROI 3–5) were located in muscle regions where no fat infiltration was observed. A representative image with selected ROIs is shown in Fig 3A. The reliability test of *T*_*2*_ measurements was performed by using a one-way analysis of variance (ANOVA) with Bonferroni correction test to accomplish pairwise comparisons of the data. An overall increase in *T*_*2*_ relaxation time was observed in all selected muscle areas in *lepb*^*-/-*^ zebrafish as compared to control fish (Fig 3B). However, the increase in *T*_*2*_ was more significant in regions 1 & 2. As the adipose tissue has in general longer *T*_*2*_ as compared to muscle [43], a significant increase in *T*_*2*_ in ROI1 and ROI2 areas in *lepb*^*-/-*^ zebrafish may be associated with an increase in fat infiltration in these muscle areas. On the other hand, areas like ROI3-ROI5, which did not show visible fat infiltration, also present a moderate increase in *T*_*2*_ values in *lepb*^*-/-*^ as compared to control zebrafish, although to a lesser extent. The *T*_*2*_ increase in *lepb*^*-/-*^ zebrafish muscles was observed in both sex, however, the extent of *T*_*2*_ increase in fat infiltered regions was slightly higher in female than in male *lepb*^*-/-*^ zebrafish. These results are in line with an overall higher fat infiltration observed in female than male *lepb*^*-/-*^ zebrafish (Fig 1). A direct relationship between tissue fat content and *T*_*2*_ relaxation time has also been found in earlier studies [44].

**Fig 4:**
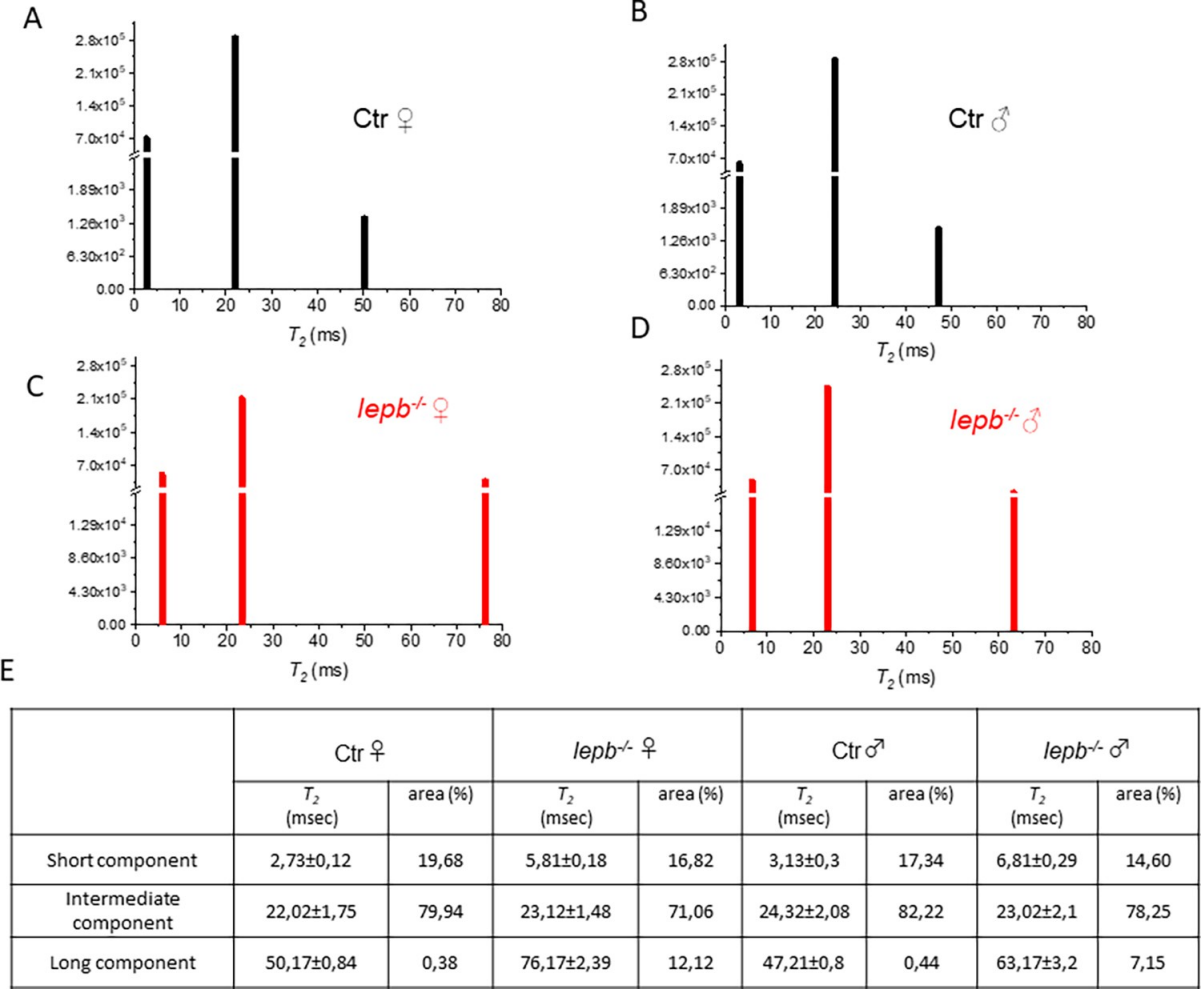
Multicomponent analysis of the experimental *T*_*2*_ relaxation decay curves using a non-negative least square based algorithm (NNLS). NNLS least-squares spectra of (A) Ctr ♀ (B) Ctr ♂ (C) *lepb*^-/-^ ♀ and (D) *lepb*^-/-^ ♂ zebrafish in ROIs located in the fat infiltered muscle area. (E) Time and amplitude of various *T*_*2*_ components (short, intermediate, and long) in msec ± SD and the percentage of areas for each component.

The *T*_*2*_ relaxation time values are significantly influenced by the mobility of water molecules associated with different effective tissue compartments, which are defined by intracellular and extracellular macromolecular components. Thus, each of these compartments exhibits distinct *T*_*2*_ value [45]. To detect, characterize and quantitatively measure multiple water compartments associated with tissue microstructures in normal and pathologic tissue, multiexponential *T*_*2*_ analysis has been successfully applied in earlier studies [35, 36, 45–48]. In the context of the present study, this approach may provide an insight into water compartmentation in muscles and how they are influenced as a result of leptin deficiency. This multicomponent analysis of the experimental *T*_*2*_ relaxation decay curves was performed using the non-negative least squares (NNLS) algorithm, which enables yielding values for the *T*_*2*_ position and relative size of each component. Results for multicomponent analysis of the experimental decay curves are presented in Fig 4. The *T*_*2*_ values of defined ROIs placed in fat infiltered muscle areas of both control and *lepb*^*-/-*^ male and female zebrafish show three well-distinguished components, centered at short, intermediate and long components and shown in Fig 4A–4D. The table in Fig 4E shows detailed values of *T*_*2*_ components as well as the percentage contribution of each individual component.

The amplitude of the short *T*_*2*_ component, calculated with NNLS algorithm, is lower in muscles of *lepb*^*-/-*^ fish as compared to control fish, suggesting a decrease in the protein bound water that may be due to muscle degeneration/atrophy in *lepb*^*-/-*^ fish. The intermediate *T*_*2*_ component can be assigned to the intra or extracellular water [45]. In our study, we see that the intermediate *T*_*2*_ component has the largest contribution to the signal in the muscle region. Signal intensity, calculated with NNLS algorithm, of this component was mainly reduced in muscles of *lepb-/-* fish as compared to control fish. These results suggest that microstructural changes in the *lepb*^*-/-*^ fish muscle may also have been linked with loss of intra or extracellular water fractions. A significant increase in the amplitude and the position of the long *T*_*2*_ component was also observed in *lepb*^*-/-*^ muscle as compared to control. Long *T*_*2*_ components in control zebrafish (50,17±0,84 ms in female and 47,21±0,80 in male) constitute only ∼0.4% of the fraction. However in *lepb*^*-/-*^ zebrafish (76,17±2,39 ms in female and 63,17±3,2 ms in male) constitute 7–12% of the fraction. The higher amplitude of this slow relaxing component in *lepb*^*-/-*^ fish muscle may reflect membrane leakiness, inflammatory changes and/or fat infiltration in the muscle. However, a shift in its position to a higher value indicates that it is likely to be associated with a higher degree of fat infiltration. There are two pools of lipids within skeletal muscle: extramyocellular lipids localized in adipose cells in between myofibers and intramyocellular lipids located within muscle cells [49, 50]. Accumulation of extramyocellular lipids such as intermuscular adipose tissue in obese patients is positively correlated with insulin resistance and reduced muscle performance [51, 52]. It is likely that extramyocellular lipids accumulation in *lepb*^*-/-*^ fish may have contributed to the increase in long *T*_*2*_ components observed in the present study. The shift in the position of long *T*_*2*_ components is more severe in female than in male *lepb*^*-/-*^ fish. These results are in line with higher fat infiltration in female seen in Figs 1 & 2. Our results show that the discrimination of healthy vs. fat-infiltrated muscle tissues may be possible through examining the changes in *T*_*2*_ multicomponent features.

**Fig 5:**
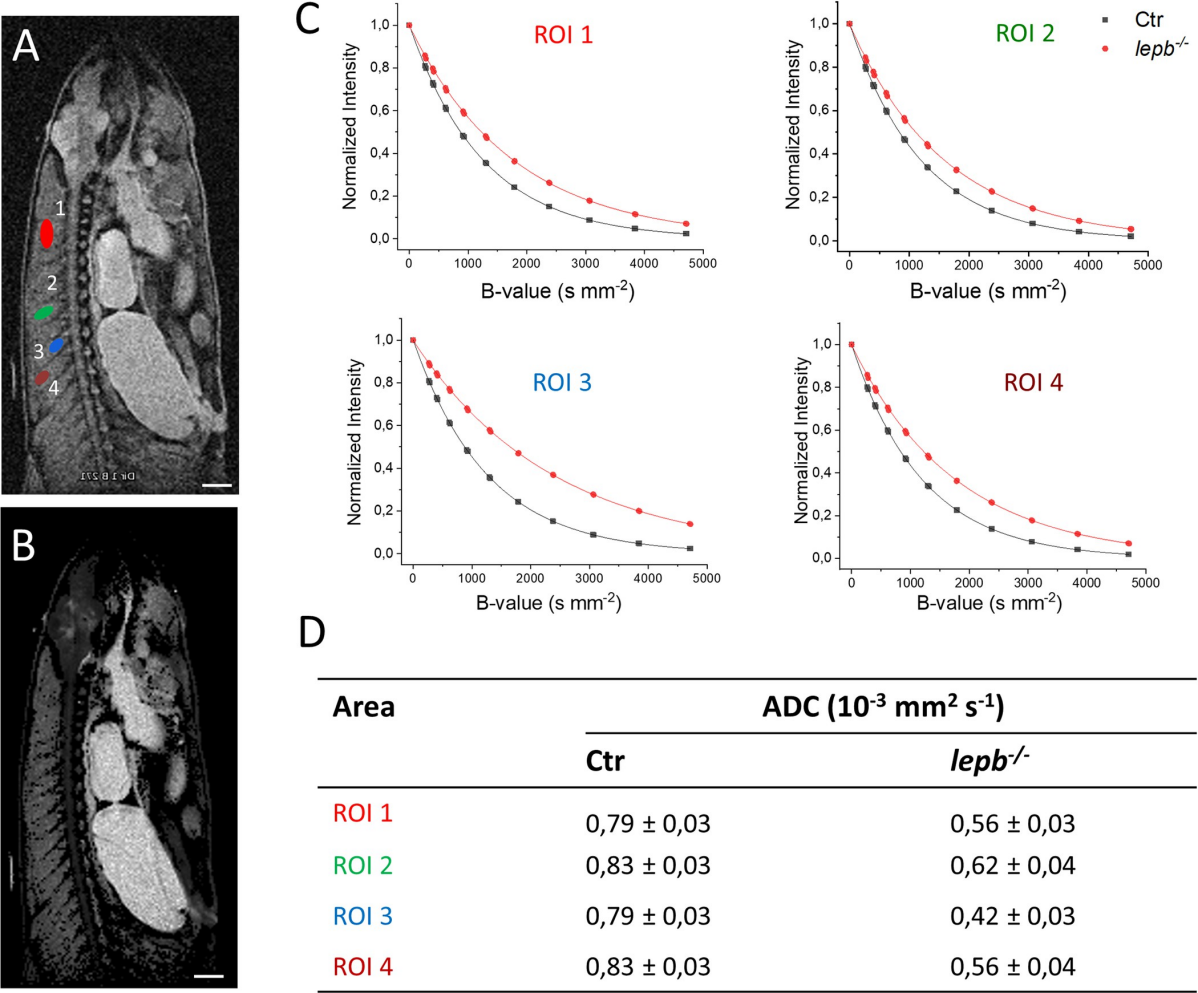
Diffusion-weighted imaging in muscle regions of control (Ctr) and *lepb*-deficient (*lepb*^*-/-*^) adult zebrafish. Diffusion measurements were performed using a spin-echo pulse sequence containing a pair of mono-polar diffusion-sensitising gradients (TR, 1000 ms; TE, 60,2 ms; diffusion gradient duration 4 ms and gradient separation of 50 ms; effective B-values range: 221, 267, 399, 614, 914, 1299, 1792, 2385, 3070, 3846, 4714 s/mm^2^). (A) A diffusion image of control zebrafish showing regions of interest (ROI) located in various muscle regions of adult zebrafish. The first ROI (red) is located in the middle of the upper myotome. The second one (green) covers the middle part of one of the myotomes. The next one (blue) is a smaller region located in the part of the myotome near the spinal cord. The last region (brown), area 0,15 ± 0,01 mm^2^ is located far from spinal cord (closer to skin). (B) Representative ADC map image of Ctr zebrafish generated through Bruker internal ‘*dtraceb*’ algorithm. It shows the distribution of ADC values where higher ADC appears bright and lower ADC appears darker. (C) Signal decay curve in respective ROIs is shown in (A). (D) Table of calculated ADC values for regions of interest is shown in (A). Scale bar: 1 mm.

Microstructural changes in muscles such as inflammation, cell swelling, atrophy &/or adiposity can lead to diffusion restrictions. Diffusion MRI is a well-established tool for non-invasive investigation of microstructural changes in muscle [42]. Apparent diffusion coefficients has been successfully used to probe disease-related structural change and the intracellular diffusional barriers in the skeletal muscles [53, 54]. To determine correlations between fat infiltration and diffusion dynamics in muscles of *lepb*^*-/-*^ fish, the intrinsic apparent diffusion coefficients (ADC) in muscles of control and *lepb*^*-/-*^ zebrafish were assessed with a series of images captured with incrementally increasing diffusion sensitizing gradient strengths (Fig 5). Diffusion properties in selected muscle regions, shown in Fig 5A, was quantified by calculation of apparent diffusion coefficients (ADC) at each voxel position in the ADC map, which shows the distribution of ADC values all over the image slice (Fig 5B) and indicates the freedom of movement for the molecules inside the different regions. A normalized mono-exponential fitting model for each selected region of interest (ROI), in muscle regions, is shown in Fig 5C. Interestingly, in the muscle areas in control zebrafish, the signal diminished more strongly than in *lepb*^*-/-*^ fish at all the b-values (Fig 5C). Remarkably, fat-infiltered muscle regions (ROI3) in *lepb*^*-/-*^ zebrafish exhibited low dephasing under strong gradients. This implies that diffusion is restricted in fat infiltered regions in *lepb*^*-/-*^ zebrafish, which was not observed in control fish where no fat infiltration was seen and, therefore, the protons move more freely in the muscle tissues. As summarized in Fig 5D, the muscle areas of *lepb*^*-/-*^ zebrafish exhibit on average lower ADC (~0.4–0.6 x 10^−3^ mm^2^ s^-1^) as compared to control muscles (~0.8 x 10^−3^ mm^2^ s^-1^).

**Fig 6:**
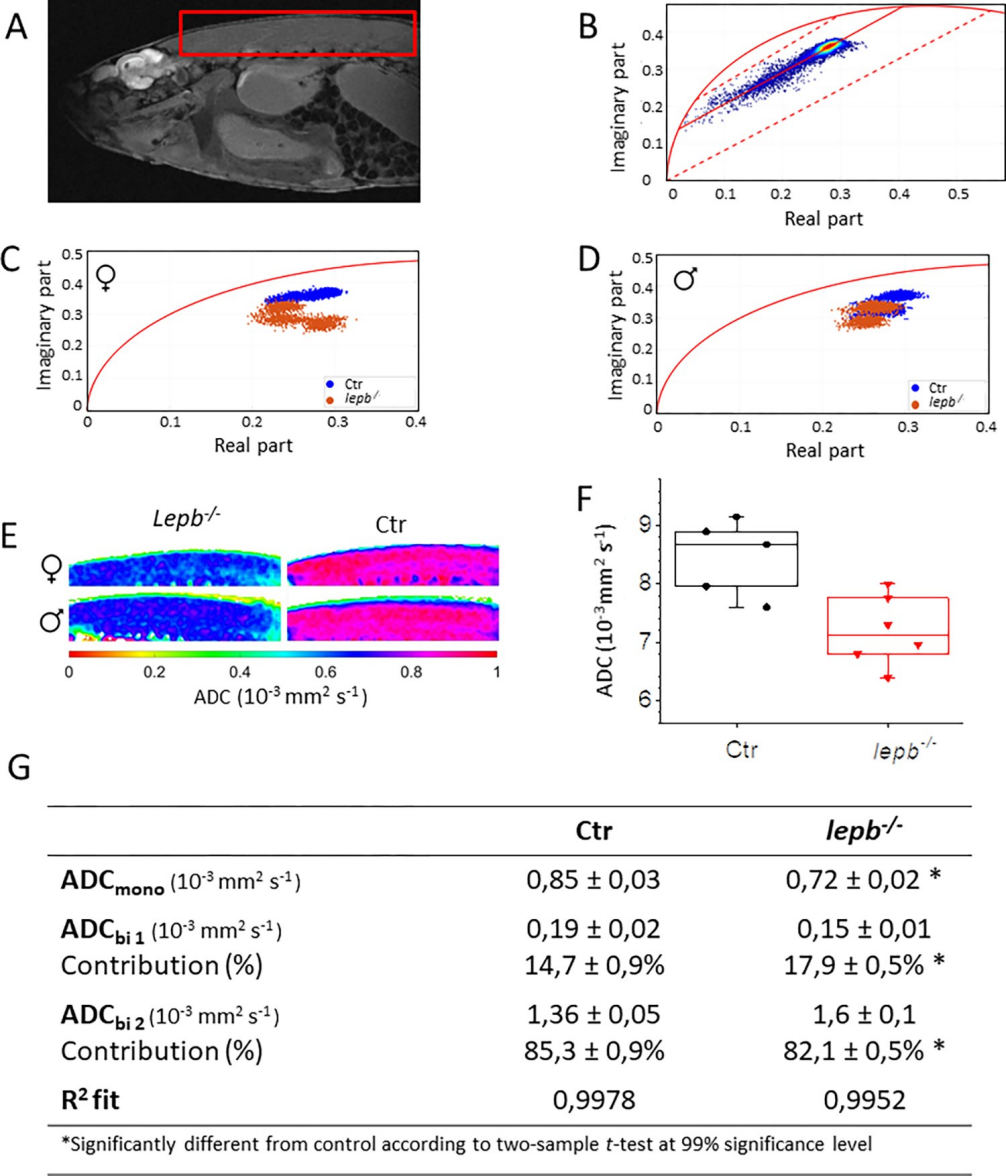
Phasor analysis of multislice 2D quantitative diffusion MRI datasets. (A) A diffusion image of a leptin-deficient (*lepb*^*-/-*^) zebrafish showing the region of interest (ROI), covering the muscle area. Voxels located outside the zebrafish were removed from the data-set by an intensity threshold. (B) Diffusion data from the ROI was transformed to phasor plot coordinates. Reference mono-component ADC values are located on the semi-circle (curved solid red line), ADC = 0 mm^2^ s^-1^ is located at [0, 0] and increases non-linearly clock-wise. General shape indicates a multi-component diffusion system, more specifically a bi-component system. ADC components were determined by line fitting (solid red line) between specified borders (dotted red lines). (C) For smaller ROI’s in the muscle area, phasor coordinates are plotted to compare ADC in female and (D) male *lepb*^*-/-*^ zebrafish with respective control (Ctr) zebrafish. (E) Mono-component ADC maps were created for female (♀**)** and male (**♂**) *lepb*^*-/-*^ and Ctr zebrafish. (F) Quantification of ADC_mono_ in Ctr and *lepb*^*-/-*^ zebrafish. (G) Table of calculated ADC values in muscle area.

To perform mono- and multi-component analysis of diffusion MRI data, phasor plot analysis was applied. Originally developed and applied for extracting multi-component half-life times in optical microscopy [55], phasor plot method was first applied to MRI data by Vergeldt *et al*. [37]. Compared to traditional line fitting, the phasor approach presents several benefits. First, as phasor transformation is performed on each voxel individually, the homogeneity of the ROI, as well as potential outliers, can be observed. Second, by combining the phasor plots of several ROI in the same plot, a fast comparison can be easily performed. Third, the phasor plot provides a quick first impression of the number of components in the ROI and the values of these components. While untangling the diffusion signal using phasor transform has been successfully used in previous work [37, 56], its application to differentiate diffusion characteristics in control and diseased animal model has not been attempted so far. Here, we present first results of comparison of the diffusion data of *lepb*^*-/-*^ and control zebrafish analyzed by phasor plot approach (Fig 6).

A representative phasor plot of *lepb*^*-/-*^ of selected muscle region (Fig 6A) is shown in Fig 6B. As shown in this figure, diffusion is described by at least two ADC components per voxel as all phasor plots are located inside, and not on top of, the semi-circle (Fig 6B). To exclude sex-specific differences, both male and female zebrafish were analysed separately. Phasor plot data of female *lepb*^*-/-*^ appear well separated from their controls (Fig 6C). For male zebrafish, some overlap is observed between the groups indicating less separation between the groups (Fig 6D). The selected muscle tissue of all replicates within a group appears to be relatively homogeneous, with no suspected outliers (Fig 6C and 6D). Mono-component ADC maps were created for both female and male *lepb*^*-/-*^ and control zebrafish as shown in Fig 6E. Based on these maps a significant difference between *lepb*^*-/-*^ and control appeared (Fig 6E and 6F). Mono-component (ADC_mono_) analysis of muscle tissue showed a significant difference (P < 0.01) between *lepb*^*-/-*^ and control zebrafish (Fig 6E and 6G). However, no significant sex-specific difference was observed (Fig 6E). This indicates overall a lower diffusion in the muscle tissue of *lepb*^*-/-*^ zebrafish compared to the control. As clear in Fig 6B, the muscle tissues show multi-component ADC systems. Therefore, low of the magnitude of diffusion seen in *lepb*^*-/-*^ could be explained by either (1) increased occurrence of low value ADC component(s), (2) decreased occurrence of high value ADC component(s), (3) decreased absolute value of ADC component(s), (4) additional low value ADC component(s), or (5) less high value ADC component(s).

Since the phasor plot of the entire muscle area showed a trend indicating a bi-component system (Fig 6B), an estimation of the individual ADC components contributing to the observed trend was performed. For this line fitting through the phasor plot points was applied for each zebrafish individually within the regions bordered by the dashed lines in Fig 6B. The component 1 (ADC_bi 1_) was fitted within 0–0.3 · 10^−3^ mm^2^/s and component 2 (ADC_bi 2_) was between 1· 10^−3^–3 · 10^−3^ mm^2^/s. The average ADC_bi1_ and ADC_bi2_ obtained for *lepb*^*-/-*^ and control fish (male and female combined) are shown in Fig 6G. Besides the value of the two ADC_bi_ components (ADC_bi 1_ and ADC_bi 2_), their individual contribution to the total signal is presented, where ADC_bi1_ + ADC_bi2_ = 100%. A significant difference (*p* = 0.01) was found between the ratio of ADC_bi 1_ and ADC_bi 2_ between *lepb*^*-/-*^ and control. These ratios, calculated from the contribution of the two ADC_bi_ values, are 0.21 and 0.17 for *lepb*^*-/-*^ and control, respectively. For confirmation, the obtained phasor plot results were subjected to conventional non-linear bi-exponential fitting. The obtained R^2^ indicate a good fit of the obtained results to the expected exponential function. These results indicate that alterations in diffusion behaviour are contributed by variation in individual ADC components of a bi-component system which reflects microstructural variations in muscles of *lepb*^*-/-*^ zebrafish as compared to controls. The results presented in this work were performed on fixed fish. It is important to note that variation in fixation procedure could affect the quantitation of diffusion. Therefore, a strict fixation protocol was implemented for both control and *lepb-/-* fish to minimize any errors that may arise due to differences in the fixation protocol.

In summary, our results suggest that lepb deficiency causes muscle adiposity in zebrafish model and further provide a brief appraisal of microstructure alterations in muscles of *lepb*^*-/-*^ zebrafish model. These results also demonstrates that μMRI in combination with novel quantitative image processing tools provides an effective and sensitive means to non-invasively study the microstructural changes in the muscles of diseased zebrafish model. While the MRI studies in this work were carried out *ex-vivo* on fixed zebrafish, in future these methods can be implemented to live fish to examine microstructural changes *in vivo*, not only in muscles but also in other relevant structures such as the brain, by utilizing various zebrafish models of human diseases.

## Supporting information

S1 File (DOCX)

S2 File (PDF)

S3 File (PDF)
