# Supplementary material for: Probing microstructural changes in muscles of leptin-deficient zebrafish by non-invasive ex-vivo magnetic resonance microimaging
Source: PLoS One. 2023 Apr 14;18(4):e0284215. doi: 10.1371/journal.pone.0284215 (PMC10104282; doi:10.1371/journal.pone.0284215)
Supplement: S1 File — (DOCX) [file pone.0284215.s001.docx]

**Supporting Information**


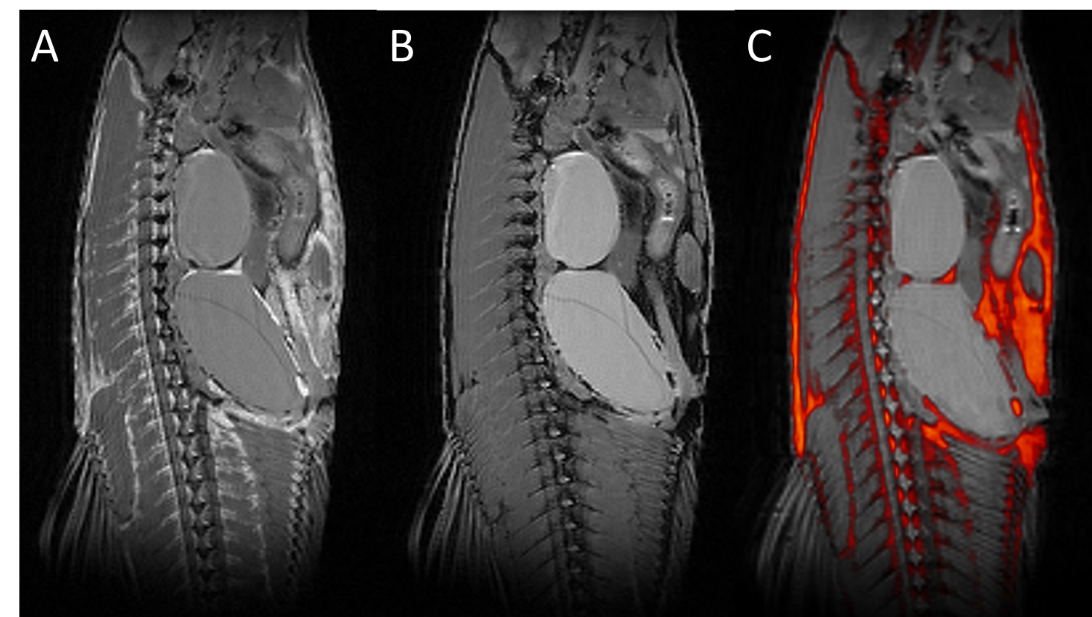


**Supplementary Fig. 1.** Representative images through muscle area of *lepb^-/-^* zebrafish measured at 750 MHz magnet. **[A]** Sagittal 2D RARE image without fat suppression; **[B]** Sagittal 2D RARE image with fat suppression; **[C]** Fat signal obtained by 3D Chemical shift selective imaging (red colored) overlayed on 3D RARE (gray scale), showing fiat infiltration in the muscles.

Data acquisition and processing were performed using Paravision 360 v3.3.

Acquisition details: 2D RARE images; TE 3.6 ms, TR 2500 ms, NA 16, RARE factor 4, Nslices 20, Image size 256 X 256, FOV 20 X 20 mm, Slice thickness 250 μm, resolution 78 X 78 X 250 μm. Acquisition details 3D RARE images; TE 5.3 ms, TR 2000 ms, NA 4, RARE factor 8, Image size 256 X 128 X 128, FOV 25 X 12.5 X 12.5 mm, resolution 98 X 98 X 98 μm. Acquisition details 3D CSSI image; TE 5.3 ms, TR 1000 ms, RARE factor 4, Shift selective excitation frequency 750.2968370 MHz (1.437 ppm, -2452.50 Hz from water signal) Excitation Pulse Bandwidth 2000 Hz, Image size 256 X 128 X 128, FOV 25 X 12.5 X 12.5 mm, resolution 98 X 98 X 98 μm.

**Supplementary Table 1:** Comparison of *T_2_* values in various muscle regions of adult zebrafish measured using multi-echo spin sequence with one slice (0.2 mm) vs. multi-slices (four slice; 0.2 mm each with a 0.2 mm interslice distance). Regions (ROIs) are same as depicted in Fig. 3A. Values are mean ± SE of the mean (n=6). *No statistically significant difference was observed between *T_2_* values measure with SS vs MS.

| **ROIs** | ***T_2_* (ms)** | |  |
| --- | --- | --- | --- |
|  | Single-slice (SS) | Multi-slice (MS) | P value*  (SS vs MS) |
| 1 | 22.66±0.37 | 22.63±0.36 | 0.94 |
| 2 | 23.86±0.11 | 23.81±0.09 | 0.72 |
| 3 | 21.23±0.15 | 21.21±0.15 | 0.94 |
| 4 | 22.71±0.44 | 22.58±0.41 | 0.83 |
| 5 | 23.20±0.27 | 23.19±0.25 | 0.96 |
