# Supplementary material for: Probing microstructural changes in muscles of leptin-deficient zebrafish by non-invasive ex-vivo magnetic resonance microimaging
Source: PLoS One. 2023 Apr 14;18(4):e0284215. doi: 10.1371/journal.pone.0284215 (PMC10104282; doi:10.1371/journal.pone.0284215)

Body-Fat_Statistic

ANOVAOneWay (2/11/2023 15:07:2

Descriptive Statistics

	N Analysis	N Missing	Mean	Standard Deviation	SE of Mean
CtrFemale	6	1	5.59786	0.73588	0.30042
LepFemale	6	1	9.47279	0.95238	0.38881
CtrMale	6	1	4.31227	0.82572	0.3371
LepMale	6	1	7.31469	1.16077	0.47388

One Way ANOVA

Overall ANOVA

	DF	Sum of Squares	Mean Square	F Value	Prob>F
Model	3	89.87747	29.95916	34.45825	4.28176E-8
Error	20	17.38867	0.86943		
Total	23	107.26614			

Null Hypothesis: The means of all levels are equal.
Alternative Hypothesis: The means of one or more levels are different.
At the 0.05 level, the population means are significantly different.

Fit Statistics

	R-Square	Coeff Var	Root MSE	Data Mean
	0.83789	0.1397	0.93243	6.6744

Means Comparisons

Bonferroni Test

	MeanDiff	SEM	t Value	Prob	Alpha	Sig	LCL	UCL
LepFemale CtrFemale	3.87493	0.53834	7.19791	3.43678E-6	0.05	1	2.29914	5.45072
CtrMale CtrFemale	-1.28559	0.53834	-2.38806	0.1615	0.05	0	-2.86138	0.2902
CtrMale LepFemale	-5.16052	0.53834	-9.58597	3.84645E-8	0.05	1	-6.73631	-3.58473
LepMale CtrFemale	1.71684	0.53834	3.18912	0.02766	0.05	1	0.14105	3.29262
LepMale LepFemale	-2.1581	0.53834	-4.00879	0.00414	0.05	1	-3.73389	-0.58231
LepMale CtrMale	3.00243	0.53834	5.57718	1.11094E-4	0.05	1	1.42664	4.57822

Sig equals 1 indicates that the difference of the means is significant at the 0.05 level.
Sig equals 0 indicates that the difference of the means is not significant at the 0.05 level.

Homogeneity of Variance Test

Levene's Test(Absolute Deviations)

	DF	Sum of Squares	Mean Square	F Value	Prob>F
Model	3	0.46002	0.15334	0.78716	0.51515
Error	20	3.89606	0.1948		

At the 0.05 level, the population variances are not significantly different.

Box Charts

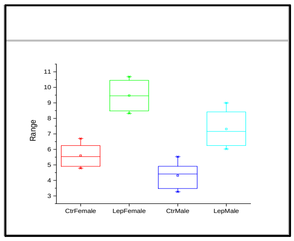

Muscle-Fat_Statistic

ANOVAOneWay (2/11/2023 15:11:2

Descriptive Statistics

	N Analysis	N Missing	Mean	Standard Deviation	SE of Mean
CtrFemale	6	1	3.09786	0.34352	0.14024
LepFemale	6	1	4.73122	0.44315	0.18091
CtrMale	6	1	2.72992	0.37816	0.15438
LepMale	6	1	4.41386	0.44234	0.18059

One Way ANOVA

Overall ANOVA

	DF	Sum of Squares	Mean Square	F Value	Prob>F
Model	3	17.215	5.73833	35.14756	3.6312E-8
Error	20	3.26528	0.16326		
Total	23	20.48028			

Null Hypothesis: The means of all levels are equal.
Alternative Hypothesis: The means of one or more levels are different.
At the 0.05 level, the population means are significantly different.

Fit Statistics

	R-Square	Coeff Var	Root MSE	Data Mean
	0.84056	0.10794	0.40406	3.74321

Means Comparisons

Bonferroni Test

	MeanDiff	SEM	t Value	Prob	Alpha	Sig	LCL	UCL
LepFemale CtrFemale	1.63336	0.23328	7.00159	5.14177E-6	0.05	1	0.95051	2.31621
CtrMale CtrFemale	-0.36794	0.23328	-1.57723	0.78258	0.05	0	-1.05079	0.31491
CtrMale LepFemale	-2.0013	0.23328	-8.57882	2.33802E-7	0.05	1	-2.68415	-1.31845
LepMale CtrFemale	1.316	0.23328	5.64119	9.6255E-5	0.05	1	0.63315	1.99885
LepMale LepFemale	-0.31736	0.23328	-1.3604	1	0.05	0	-1.00021	0.36549
LepMale CtrMale	1.68394	0.23328	7.21842	3.29615E-6	0.05	1	1.00109	2.36679

Sig equals 1 indicates that the difference of the means is significant at the 0.05 level.
Sig equals 0 indicates that the difference of the means is not significant at the 0.05 level.

Homogeneity of Variance Test

Levene's Test(Absolute Deviations)

	DF	Sum of Squares	Mean Square	F Value	Prob>F
Model	3	0.05062	0.01687	0.39713	0.7565
Error	20	0.84976	0.04249		

At the 0.05 level, the population variances are not significantly different.

Box Charts

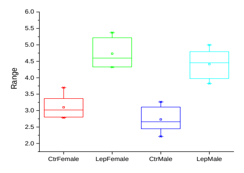

Supplement: S2 File — (PDF) [file pone.0284215.s002.pdf]
